# The Effect of Timing and Methods for the Diagnosis of Gestational Diabetes Mellitus on Obstetric Complications

**DOI:** 10.3390/medicina59050854

**Published:** 2023-04-28

**Authors:** Gintarė Galdikaitė, Atėnė Simanauskaitė, Gitana Ramonienė, Eglė Savukynė, Laura Malakauskienė, Viktorija Tarasevičienė

**Affiliations:** 1 Faculty of Medicine, Lithuanian University of Health Sciences, 44307 Kaunas, Lithuania; 2 The Department of Obstetrics and Gynecology, Lithuanian University of Health Sciences Hospital Kauno Klinikos, 50009 Kaunas, Lithuania

**Keywords:** gestational diabetes mellitus, diagnosis, complications

## Abstract

*Aim.* To compare the impact of the time and method of diagnosis on gestational diabetes mellitus (GDM) in women who gave birth at the Hospital of the Lithuanian University of Health Sciences (LUHS) Kauno klinikos. *Methods.* A retrospective study was performed using data from the Department of Obstetrics and Gynecology of the LUHS Birth Registry to analyze the data of women who gave birth and had GDM in 2020–2021. The subjects were divided based on the type of diagnosis: GDM was diagnosed either at the first antenatal visit when fasting plasma glycemia (FPG) was ≥5.1 mmol/L (early diagnosis group) or after OGTT at 24 + 0 − 28 + 6 weeks of gestation when at least one pathological glycemic index was observed: fasting glycemia 5.1–6.9 mmol/L or 1-h glycemia ≥10.0 mmol/L or 2 h glycemia 8.5–11.0 mmol/L (late diagnosis group). The results were processed using IBM SPSS. *Results*. The early diagnosis group had 1254 (65.7%) women, the late diagnosis group had 654 (34.3%). More primigravida women were in the late diagnosis group (*p* = 0.017) while more multigravida were in the early diagnosis group (*p* = 0.033). The early diagnosis group had more obese women (*p* = 0.001), including those with a BMI > 40 (*p* = 0.001). In the early diagnosis group, GDM was more frequently diagnosed in women who gained <11 kg (*p* = 0.005), while in the late diagnosis group—>16 kg (*p* = 0.001). FPG was higher in the early diagnosis group (*p* = 0.001). Glycemia was more commonly corrected with lifestyle changes in the late diagnosis group (*p* = 0.001), and with additional insulin therapy in the early diagnosis group (*p* = 0.001). Polyhydramnios and preeclampsia were more common in the late diagnosis group (*p* = 0.027 and *p* = 0.009). There were more large-for-gestational-age neonates in the late diagnosis group (*p* = 0.005). Macrosomia was more common in the late diagnosis group (*p* = 0.008). *Conclusions.* GDM is more commonly diagnosed with OGTT in primigravida women. Higher pregestational weight and BMI has an impact on the early diagnosis of GDM and need for insulin therapy with lifestyle changes. Late diagnosis of GDM is connected with obstetric complications.

## 1. Introduction

Gestational diabetes mellitus (GDM) is a serious complication that occurs during pregnancy and is characterized by a state of carbohydrate intolerance resulting in varying degrees of hyperglycemia with onset or first recognition during pregnancy [1,2,3]. It is the most commonly diagnosed endocrinopathy and its incidence is increasing every year [4,5]. In 2017, hyperglycemia in pregnancy affected 21.3 million births worldwide (16.2%), with 86.4% of cases attributed to GDM [6]. The prevalence of GDM varies from 2 to 40% in different countries [3]. A meta-analysis conducted in 2017 reported that the prevalence of GDM in Europe is approximately 5.4%. However, estimating the prevalence of GDM is difficult because rates vary from study to study due to the lack of universally accepted diagnostic criteria and differences in screening procedures [7].

The issue of hyperglycemia during pregnancy and the diagnostics of GDM were brought to light by the Hyperglycemia and Adverse Pregnancy Outcomes (HAPO) study in 2008 [8]. The HAPO study provided an opportunity to revise the diagnostic criteria for GDM. The proposed criteria for 75 g, 2 h OGTT are that any one or more of the thresholds be met or exceeded: fasting plasma glucose (FPG) of 5.1 mmol/L or 1 h plasma glucose 10.0 mmol/L or 2-h plasma glucose 8.5 mmol/L [6,8,9]. This study found a significant link between even slight maternal hyperglycemia and adverse outcomes such as fetal macrosomia, fetal hyperinsulinemia and hyperglycemia, preeclampsia, Caesarean section (CS), premature birth, dystocia, clinically significant neonatal hypoglycemia, hyperbilirubinemia, and more frequent treatment of neonates in the Intensive Care Unit [6,7,8,10]. 

Before the publication of the HAPO study, GDM was diagnosed by using two-step method OGTT at 24–28 gestational weeks, and it was only performed in pregnant women with risk factors. However, it was not suitable and accurate during pregnancy due to physiological metabolic changes occurring in the pregnant body [8]. According to the HAPO study, in 2010 The International Association of Diabetes and Pregnancy Study Groups (IADPSG) and in 2013 the World Health Organization (WHO) published new universal screening criteria of GDM: the first antenatal visit should include an FPG test and a 75 g OGTT, which is performed between 24 + 0 and 28 + 6 weeks of gestation in pregnant women who are not diagnosed with GDM at the first antenatal visit. FPG ≥ 5.1 mmol/L in early pregnancy (up to 24 weeks) is also recommended to be classified as GDM by the IADPSG and WHO [11,12]. FPG test identifies early hyperglycemia, which could be caused by both GDM and diabetes mellitus. Early diagnosis and treatment of hyperglycemia lead to a lower risk of adverse obstetrical outcomes [13,14]. However, some studies suggest that the early treatment of GDM in early pregnancy show conflicting results. They state that the early treatment, compared to later in the pregnancy, does not always translate to improved obstetrical outcomes [15]. Bianchi et al. showed that women diagnosed and treated for early-onset GDM were more prone to be insulin-treated during pregnancy but showed no differences in neonatal outcomes such as small-for-gestational-age neonates, CS, macrosomia, and large-for-gestational-age neonates (LGA) [16].

The national screening program for GDM in Lithuania was initiated in 2018, following the methodology recommended by WHO [2,10]. According to the Institute of Hygiene, the incidence of GDM has been increasing in Lithuania every year due to the new diagnostic criteria. In 2015 it was about 2%, in 2018 it increased to 6%, and in 2021, it reached a staggering 22% [17]. The HAPO study not only led to changes in GDM’s diagnostic criteria but also contributed to the higher prevalence of GDM worldwide and in Lithuania [18].

Early diagnosis and treatment of GDM are crucial to decrease the risk of obstetrical complications both in the early and late stages of pregnancy [14,19]. Women with GDM and their children are at a higher risk of developing obesity, cardiovascular diseases, type 2 diabetes mellitus, or even metabolic syndrome in the future [4,19].

Treatment of GDM begins immediately after diagnosis and can include several methods of treatment: by changing lifestyle habits (dietary and physical activity modification) or lifestyle changes with additional insulin therapy [20]. Dietary modifications involve consuming a balanced diet with controlled carbohydrate intake, choosing low glycemic index foods, and avoiding sugary and processed foods. Physical activity modifications involve regular exercise, which can improve insulin sensitivity and help regulate blood glucose levels. Women with GDM are advised to engage in moderate-intensity exercise for at least 30 min a day [6]. Studies suggest that early diagnosis and proper insulin treatment could decrease the risk of perinatal complications [21].

The study aims to evaluate and compare the relationship between GDM diagnosis time and method in women who gave birth at the Department of Obstetrics and Gynecology in the Hospital of the Lithuanian University of Health Sciences Kauno klinikos. 

## 2. Materials and Methods

A retrospective study was conducted at the Department of Obstetrics and Gynecology of LUHS Hospital Kauno klinikos, where data were obtained from the Birth Registry of women who had GDM and gave birth between 1 January 2020, and 31 December 2021. Data were collected from the Birth Registry database and maternal care log book. Approval from The Kaunas Regional Biomedical Research Ethics Committee Nr. BE-2-67 was obtained.

From the maternal log book, socio-demographic data and obstetrical history of woman were collected and evaluated, including age, education, marital status (married, partnership, other (single, divorced, or widowed), number of prior pregnancies and births, gestational age at delivery, and unfavorable obstetrical history for the reason of collecting general information of the studied groups and possible associations to the studied issue. Maternal height and pre-pregnancy weight were also assessed, and body mass index (BMI) was calculated. A BMI of 18.5–24.9 kg/m^2^ was considered normal, while obesity was defined as BMI ≥ 30 kg/m^2^ and distributed into three classes: I—30–34.9 kg/m^2^, II—35–39.9 kg/m^2^, III > 40 kg/m^2^ [22]. Gestational weight gain (GWG) during pregnancy was calculated by subtracting the weight before pregnancy or at the first antenatal visit (recorded in the maternal care log book) from the weight before delivery (recorded in the birth records or in the maternal care log book at the last antenatal visit). GWG was classified into several groups: for women with BMI < 18.5 kg/m^2^, the recommended total GWG was 12.5–18 kg, for women with a normal BMI—11.5–16 kg, and for obese women, it was 5–9 kg [23].

The primary outcome of this study was to determine the frequency of a different GDM diagnosis method, while obstetrical outcomes were considered to be secondary outcomes of this investigation.

The subjects were divided into two groups based on the time and method of GDM diagnosis regulated by the Lithuanian Ministry of Health: GDM diagnosed during the first antenatal visit (until 14 weeks of gestation) when FPG was ≥5.1 mmol/L (early diagnosis group), or after the OGTT performed at 24 + 0 − 28 + 6 weeks of pregnancy, when at least one pathological indicator of glycemia was found: FPG ≥5.1 mmol/L, glycemia after 1-h after drinking 75 g of glucose ≥10.0 mmol/L, glycemia after 2-h 8.5–11.0 mmol/L (late diagnosis group).

GDM was classified according to treatment based on the regulations of the Lithuanian Ministry of Health: when dietary and physical activity modification were sufficient to control blood glucose level, and when additional insulin therapy was required. 

Hypertensive conditions during pregnancy were diagnosed when arterial blood pressure was ≥140/90 mmHg on two occasions 4 h apart in a previously normotensive woman after ≥20 weeks of gestation without proteinuria (gestational hypertension) or arterial blood pressure ≥140/90 mmHg with proteinuria ≥300 mg in a 24-h urine specimen (preeclampsia, preeclampsia with severe symptoms). Chronic (primary) hypertension and chronic (primary) pregnancy-complicated hypertension were diagnosed when elevated blood pressure was found before the 20th week of gestation. Polyhydramnios was diagnosed in the presence of an excessive amount of amniotic fluid diagnosed by ultrasound when the amniotic fluid index was ≥240 mm or the single deepest pocket of amniotic fluid was ≥80 mm. Fetal macrosomia was determined if the birth weight of the fetus was ≥4000 g (regardless of the gestational age). A large-for-gestational-age neonate was defined as birthweight above the 90th percentile adjusted for gender and gestational age.

The study evaluated the course of labor, labor induction and augmentation methods, outcomes, indications for CS, Apgar score at 1 and 5 min, and the gender and weight of the neonate.

Data were collected and systematized in “Microsoft Office Excel”, processed and analyzed using the statistical data analysis package “IBM Statistics 23”. The relationship between two random samples of qualitative data was evaluated using the chi square (χ^2^) test, and the Student’s (t) test was used to compare quantitative measurements. The median (Me), minimum (min), and maximum (max) values were used to describe the interval scale variables, with the mean (m) and standard deviation (SD) also reported. The results were considered significant when *p* < 0.05. Percentage values, frequencies, and averages of continuous values with SD were calculated. To compare the odds of an event occurring in two different groups, the odds ratio was calculated.

## 3. Results

In 2020–2021, a total of 6005 women delivered at the Department of Obstetrics and Gynecology, a tertiary teaching center of the Hospital of Lithuanian University of Health Sciences, with 1908 (31.8%) of those women having GDM and included in this study. Of these, 1254 (65.7%) were in the early diagnosis group (20.9% of all who delivered), while the late diagnosis group included 654 (34.3%) patients with GDM (10.9% of all who delivered). 

When comparing maternal characteristics, the majority of patients were married and had higher education. The late diagnosis group had more primigravida women, while the early diagnosis group had more multigravida women. However, unfavorable obstetrical history did not differ significantly between the groups. Maternal characteristics are demonstrated in Table 1.

Table 2 shows unfavorable obstetrical history. Assisted human reproduction was more common in the late diagnosis group.

Table 3 shows the distribution of BMI, where the early diagnosis group had a greater average BMI, BMI 30–34.9 kg/m^2^ and BMI > 40 kg/m^2^. There were more obese women in the early diagnosis group. The average GWG in the late diagnosis group was higher, and there were significantly more patients who gained more than 16 kg. In contrast, an average GWG of less than 11 kg was found in the early diagnosis group.

FPG was significantly higher in the early diagnosis group. Hyperglycemia was more commonly corrected with dietary and physical activity modification among women in the late diagnosis group, and dietary and physical activity modification with insulin therapy in the early diagnosis group. Glycemia correction and the GDM treatment method is shown in Table 4. Obese women diagnosed with GDM during their first antenatal visit have a 2.3 times higher risk of needing insulin therapy compared to women with a normal BMI (OR 2.4, CI 1.8–3.1).

In the late diagnosis group, there were significantly higher occurrences of polyhydramnios and preeclampsia. Other pregnancy outcomes were similar in both groups, as shown in Table 5. 

Table 6 shows the indications for the CS. Breech presentation was more often as an indication for CS in the early diagnosis group. 

Large-for-gestational-age neonates and macrosomia were more often diagnosed in the late diagnosis group. Additionally, more cases of LGA neonates and macrosomia were identified when GDM was managed solely through dietary and physical activity modification in the late diagnosis group. Labor complications are outlined in Table 7.

Mechanical device for labor inductions were more frequently utilized in the early diagnosis group. However, labor induction, the gestational age when labor was induced, and other methods of induction (amniotomy, induction with misoprostol or oxytocin) were not significant. The need for urgent CS due to failed induction of labor was also not significant, as shown in Table 8. 

## 4. Discussion

The presented study is the first in Lithuania to examine the impact of timing and method of GDM diagnosis on adverse obstetrical outcomes, following the adaptation of new GDM diagnostic guidelines. Additional insulin therapy for glycemia control is more often needed when GDM is diagnosed before 20 gestational weeks. Conversely, adverse obstetrical outcomes such as polyhydramnios, macrosomia, and large-for-gestational-age neonates are more prevalent when GDM is diagnosed after 20 gestational weeks.

GDM is a common condition that affects both the mother and fetus during and after pregnancy and has long-term effects [2,13]. However, our study did not analyze the long-term outcomes for the mother and child. The new method of GDM screening was introduced by the WHO and IADPSG, and the national pregnancy screening of GDM in Lithuania was implemented only in 2018, resulting in an increasing rate of GDM every year [13,14,24,25]. Our study also confirmed the growing tendency of GDM rates, with 31.8% of women being affected in 2020–2021 [11,26].

In our study, primigravida women were more frequently diagnosed with GDM by OGTT. Similar data was reported in a study by D. Borohoonhirunsarn et al., where women in the late GDM group were more likely to be primigravida (51.7%) and younger [13]. However, most other studies do not analyze the link between obstetrical history and timing of GDM diagnosis [21,27]. A meta-analysis by Zhang Yu et al. suggested that being primigravida is associated with a lower risk of GDM [27].

Obesity before and during pregnancy increases the risk of GDM and is associated with adverse obstetrical outcomes [28]. This is mainly due to metabolic changes before the pregnancy and worsened glucose tolerance [3]. In our study, one fifth of women were obese, and they were more often in early diagnosis group. Similar findings were reported by Y. Weng et al., who investigated the link between obesity, clustering of metabolic risk factors in early pregnancy and GDM [29]. Our study and others have also observed that obese women are more frequently diagnosed with GDM using the FPG test in the first antenatal visit [18,30]. Therefore, it is crucial to educate young women about a healthy, active lifestyle and maintaining a normal body weight before pregnancy [18,28,31].

In our study, we observed not only the pregestational BMI but also the GWG. We found that the majority of women in the early diagnosis group gained less than 11 kg during pregnancy. This finding is significant because excessive GWG may also lead to increased insulin resistance and further exacerbate maternal hyperglycemia [31]. Therefore, GDM diagnosed and treated early in pregnancy can result in less weight gain. Our results are consistent with a 2017 meta-analysis and a 2020 study conducted in Italy [14,32].

Significantly higher FPG levels were found in the early diagnosis group. This emphasizes the importance of conducting FPG tests during the first antenatal visit to achieve early glycemia control and reduce the risk of related obstetrical outcomes [19]. Other studies, such as a 2017 meta-analysis by J. Immanuel et al., have also reported that women who are diagnosed with GDM before 20 gestational weeks may require insulin therapy in addition to dietary and physical activity modifications [26,29,33].

GDM is associated with several adverse obstetrical outcomes [33], including polyhydramnios, which was found more frequently in the late diagnosis group in our study. The risk of polyhydramnios increases up to 18% for patients with GDM [34,35]. However, a study performed in Iran found that polyhydramnios was more frequently associated with early-onset GDM [34]. GDM is also linked with preeclampsia as both are connected to insulin resistance [35]. Our study and another large study suggest that women in the late diagnosis group more likely to experience preeclampsia [36].

One of the most common obstetrical complications of GDM is macrosomia and large for gestational age neonates [2,11,12,35,37]. In our study, we observed that overweight fetuses were more commonly diagnosed in the late diagnosis group, which is consistent with findings from a study by Y. Wenrui et al. [37]. These complications increase the risk of trauma during labor, the need for CS, and prolonged the hospital stays in the Neonatal Intensive Care Unit [26,28]. It is therefore important to estimate fetal weight accurately during antenatal ultrasound, and a suitable birth plan should be made [38].

The appropriate timing and method of GDM diagnosis are crucial not only for preventing adverse obstetrical outcomes but also for the long-term maternal risk of developing type 2 diabetes mellitus [39]. Similar data have been reported in various other studies conducted by researchers all over the world [4,7,14,21,31]. A large cohort study by M. V. Diaz-Santana with a sample size of 50,884 demonstrated that a history of GDM significantly increases the rates of type 2 diabetes [40]. Therefore, an OGTT is recommended 6–12 weeks after pregnancy. While our study focused on adverse obstetrical outcomes, it is important to analyze long-term maternal and neonatal complications in future research.

In comparison to previous studies in this field, this novel study offers a more comprehensive analysis of GDM method of diagnosis impact on obstetrical complications. Our research is the first in Lithuania after the new GDM diagnosis guidelines. Moving forward, future studies could benefit from incorporating longitudinal data to better understand the long-term effects of the variables examined in this study. Overall, this study provides a strong foundation for future research in this area and highlights the importance of continued investigation into the factors that impact of women with GDM.

## 5. Conclusions

In summary, primigravida women are more frequently diagnosed with GDM through the use of OGTT. Obese women diagnosed with GDM during the first antenatal visit have a higher risk of needing insulin therapy. Obstetrical outcomes such as preeclampsia, polyhydramnios, fetal macrosomia and large-for-gestational-age neonates are significantly more common when GDM is diagnosed using OGTT.

## Figures and Tables

**Table 1:** Maternal characteristics.

		Early Diagnosis Group *n* = 1254	Late Diagnosis Group *n* = 654	*p* Value
Patients age		31 (15–46; 5.1)	31 (15–45; 5.3)	0.540
Education	Basic	69 (5.5)	37 (5.7)	0.444
	Secondary	325 (25.9)	172 (26.3)	0.428
	Higher	860 (68.6)	445 (68.0)	0.595
Marital status	Married	917 (73.1)	482 (73.7)	0.394
	Partnership	291 (23.2)	157 (24.0)	0.348
	Other	46 (3.67)	15 (2.3)	0.959
Obstetrical history	Primigravida	373 (29.7)	226 (34.6)	0.017
	Multigravida	881 (70.3)	428 (65.4)	0.033

Data presented as Me (min-max; mean), *p*-value—Mann–Whitney *U* test. n (%), *p*-value—χ^2^ the homogeneity of variables criteria, *p* < 0.05—statistically significant result.

**Table 2:** Unfavorable obstetrical history.

	Early Diagnosis Group	Late Diagnosis Group	*p* Value
	*n* = 1254	*n* = 654	
Spontaneous miscarriage	274 (21.9)	161 (24.6)	0.088
Previous CS	234 (18.6)	116 (17.7)	0.691
Infertility	40 (3.5)	16 (2.5)	0.829
Assisted human reproduction	19 (1.5)	32 (4.9)	0.001
Previous premature birth	44 (3.5)	16 (2.5)	0.829
Stillborn	27 (2.1)	11 (1.7)	0.766

*n* (%), *p*-value—χ^2^ the homogeneity of variables criteria. *p*-value—Mann–Whitney *U* test, *p* < 0.05—statistically significant result.

**Table 3:** Anthropometric characteristics and weight gain during pregnancy.

		Early Diagnosis Group	Late Diagnosis Group	*p* Value
		*n* = 1254	*n* = 654	
Patients’ height		168.28 (6.25)	167.36 (6.26)	0.003
Patients’ weight		75.53 (18.6)	71.79 (16.3)	0.001
Pregestational BMI		26.7 (15.7–49.6; 6.2)	25.6 (14.9–45.3; 5.3)	0.001
	<18.5	44 (3.5)	27 (4.1)	0.254
	18–24.9	565 (45.1)	333 (50.9)	0.007
	25–29.9	316 (25.2)	166 (25.4)	0.465
	30–34.9	196 (15.6)	83 (12.7)	0.034
	35–39.9	73 (5.8)	36 (5.5)	0.612
	>40	60 (4.8)	9 (1.38)	0.001
Obesity		329 (26.2)	127 (19.42)	0.003
GWG		11.5 (–14–40; 6.5)	13 (–10–40; 6.5)	0.001
	<11 kg	612 (48.8)	278 (42.5)	0.005
	11–16 kg	406 (32.4)	203 (31.04)	0.725
	>16 kg	236 (18.82)	173 (26.5)	0.001

*p*-value—Mann–Whitney U test, *p* < 0.05—statistically significant result. Data presented as Me (min-max; mean), *p*-value—Mann–Whitney *U* test. *n* (%), *p*-value—χ^2^ the homogeneity of variables criteria, *p* < 0.05—statistically significant result.

**Table 4:** Glycemia and treatment of GDM.

		Early Diagnosis Group	Late Diagnosis Group	*p* Value
		*n* = 1254	*n* = 654	
OGTT (mmol/L)		5.46 (5–9.09; 0.38)	5.2 (3.3–11.4; 0.76)	0.001
Glycemia after 1 h (mmol/L)			8.8 (3.2–16.2; 2.1)	
Glycemia after 2 h (mmol/L)			7.0 (3–18.2; 2.0)	
GD treatment	Lifestyle changes	800 (63.8)	479 (73.24)	0.001
	Additional insulin therapy	454 (36.2)	175 (26.8)	0.001

Data presented as Me (min–max, mean), *p*-value—Mann–Whitney *U* test. *p*-value—Mann–Whitney *U* test, *p* < 0.05—statistically significant result. *n* (%), *p*-value—χ^2^ the homogeneity of variables criteria.

**Table 5:** Pregnancy complications.

	Early Diagnosis Group	Late Diagnosis Group	*p* Value
	*n* = 1254	*n* = 654	
Hypertensive disorders in pregnancy	124 (9.9)	75 (11.5)	0.147
Gestational hypertension	85 (6.8)	41 (6.3)	0.666
Preeclampsia	23 (1.8)	25 (3.8)	0.009
Preeclampsia with severe features	16 (1.3)	9 (1.4)	0.428
Polyhydramnios	127 (10.1)	86 (13.2)	0.027
Premature birth	99 (7.9)	49 (7.5)	0.623

*n* (%), *p*-value—χ^2^ the homogeneity of variables criteria. *p*-value—Mann–Whitney *U* test, *p* < 0.05—statistically significant result.

**Table 6:** Pregnancy complications.

	Early Diagnosis Group	Late Diagnosis Group	*p* Value
	*n* = 1254	*n* = 654	
Repeated CS	61 (4.9)	28 (4.3)	0.721
Labor dystocia	95 (7.6)	47 (7.1)	0.622
Fetal distress	39 (3.1)	29 (4.4)	0.080
Breech position	38 (3.0)	33 (5.1)	0.020
Other	48 (3.8)	28 (4.3)	0.318

*n* (%), *p*-value—χ^2^ the homogeneity of variables criteria. *p*-value—Mann–Whitney *U* test, *p* < 0.05—statistically significant result.

**Table 7:** Labor complications.

		Early Diagnosis Group	Late Diagnosis Group	*p* Value
		*n* = 1254	*n* = 654	
Vaginal birth		969 (77.3)	490 (74.9)	0.872
Caesarean section	Total number	285 (22.7)	164 (25.1)	0.128
	Urgent	170 (13.6)	103 (15.8)	0.429
	Elective	115 (9.2)	61 (9.3)	0.456
GA at birth		39 (22–41; 2.23)	39 (24–41; 1.78)	0.267
APGAR score	1	9 (0–10; 1.44)	9 (0–10; 1.51)	0.953
	5	10 (0–10; 1.26)	10 (0–10; 1.21)	0.631
Gender	Male	651 (51.9)	360 (55.1)	0.096
	Female	603 (48.1)	294 (44.9)	0.904
Newborn weight		3404.5 (628.4)	3450.6 (608.8)	0.125
Large for gestational age	Total number	129 (10.3)	95 (14.5)	0.005
	Lifestyle changes	75 (5.98)	69 (10.6)	0.004
	Additional insulin therapy	54 (4.3)	26 (4.0)	0.635
Fetal macrosomia	Total number	162 (12.9)	112 (17.1)	0.008
	Lifestyle changes	111 (8.9)	82 (12.5)	0.008
	Additional insulin therapy	51 (4.1)	30 (4.6)	0.299
Stillbirth		10 (0.8)	7 (1.1)	0.283

Data presented as Me (min-max; mean), *p*-value—Mann–Whitney *U* test. *n* (%), *p*-value—χ^2^ the homogeneity of variables criteria. *p*-value—Mann–Whitney *U* test, *p* < 0.05—statistically significant result.

**Table 8:** Labor induction.

		Early Diagnosis Group	Late Diagnosis Group	*p* Value
		*n* = 1254	*n* = 654	
Labor induction		617 (49.2)	311 (47.6)	0.753
GA, when labor was induced		39 (22–41; 2.23)	39 (24–41; 1.78)	0.267
Method of induction	Amniotomy	293 (23.4)	167 (25.5)	0.149
	Misoprostol	222 (17.7)	102 (15.6)	0.882
	Oxytocin	52 (4.2)	28 (4.3)	0.445
	Mechanical device	50 (4.0)	14 (2.1)	0.013
CS due to failed induction of labor		134 (10.7)	76 (11.6)	0279

Data presented as Me (min-max; mean), *p*-value—Mann–Whitney *U* test. *n* (%), *p*-value—χ^2^ the homogeneity of variables criteria. *p*-value—Mann–Whitney *U* test, *p* < 0.05—statistically significant result.

## Data Availability

Anonymized data used for the study is stored on a separate biomedical study data storage computer that was used to conduct the biomedical study. Data will be stored for 15 years after the study and later destroyed.
